# A Rare Case of an Isolated Nasal Floor Abscess in an Immunocompetent Patient

**DOI:** 10.7759/cureus.97694

**Published:** 2025-11-24

**Authors:** Jun Kit Thong, Benjamin Jead Wern Ho, Eng Haw Lim, Sharanjeet Singh

**Affiliations:** 1 Otolaryngology - Head and Neck Surgery, Miri Hospital, Miri, MYS

**Keywords:** nasal abscess, nasal floor, nasal floor abscess, nasal septal abscess, nose infection

## Abstract

Nasal abscesses are uncommon and typically associated with systemic conditions, trauma, or infections of adjacent structures. Isolated nasal abscesses, particularly involving the nasal floor, are exceedingly rare, with no prior documented cases in the English medical literature. This report presents the case of a 35-year-old immunocompetent woman who developed a nasal floor abscess without identifiable predisposing factors. She presented with localized swelling, pain, and pus discharge, which were managed successfully with incision and drainage and intravenous antibiotics. The patient recovered fully with no residual complications. This case highlights the importance of considering rare anatomical sites for abscess formation in the differential diagnosis of nasal swelling and underscores the value of prompt surgical intervention and appropriate antibiotic therapy in achieving favourable outcomes.

## Introduction

Nasal abscesses are typically associated with systemic conditions causing immunosuppression, trauma, or infections of the adjacent structures [1]. Isolated nasal abscesses are infrequently reported, and cases involving the nasal floor are unprecedented in English medical literature. A search of PubMed, Scopus, and Google Scholar using the terms ‘nasal floor abscess’, ‘nasal floor infection’, and ‘nasal abscess’ revealed no prior documented cases of isolated abscesses at this anatomical site. The search primarily returned articles focusing on nasal septal abscesses, further highlighting the rarity of this presentation. This underscores the need for clinicians to consider uncommon anatomical sites in the differential diagnosis of patients presenting with localized nasal swelling.

## Case presentation

A previously healthy 35-year-old Malay woman presented to the Otorhinolaryngology clinic with a two-week history of pain and swelling over the right nasal floor. The swelling had progressively increased, extending to the right upper lip. She also reported pus discharge starting two days prior to her visit. Notably, she had no systemic signs of infection, such as fever or lethargy, and denied symptoms suggestive of sinusitis, including facial pain or toothache.

**Figure 1:**
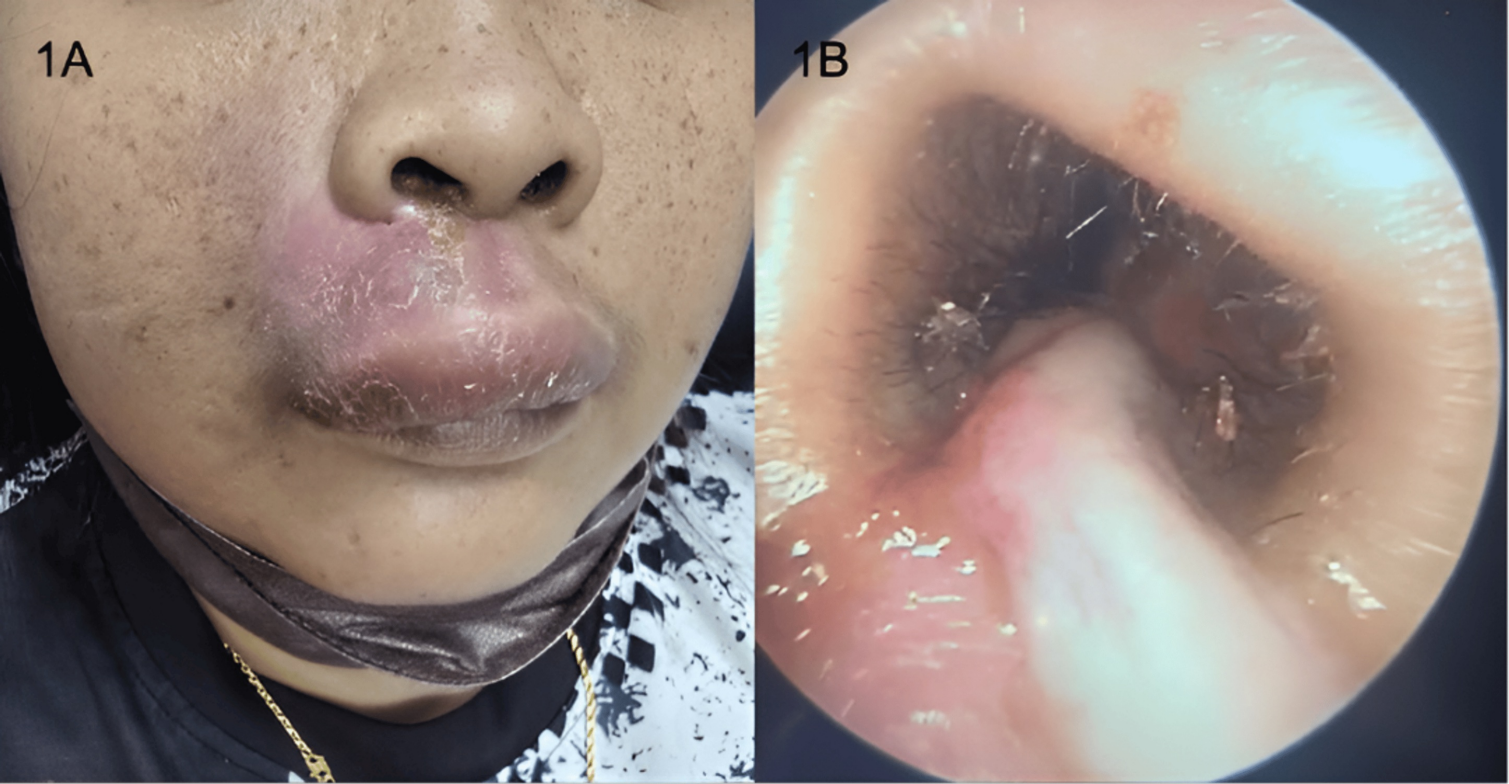
Extensive swelling and erythema of the right upper lip extending to the nasal floor (1A). Punctum with pus discharge seen on the nasal floor (1B).

On examination, there was swelling and erythema over the right upper lip extending to the nasal floor. The swelling was tense and tender on palpation. Nasoendoscopy revealed a punctum at the nasal floor, with pus discharge expressed on milking (Figures 1A, 1B). There was no evidence of a nasal septal abscess. A dental evaluation ruled out odontogenic causes, revealing normal dental findings.

The patient underwent incision and drainage under local anesthesia. The punctum was extended, allowing for the drainage of approximately 3 mL of pus. The abscess cavity was packed with gauze soaked in diluted povidone-iodine. She was admitted for intravenous ceftriaxone therapy and daily dressing changes. Pus cultures and acid-fast bacilli tests showed no significant growth. As the patient had not received any antibiotics prior to the procedure and the sample was collected before cleaning the wound with iodine, the negative culture result may reflect the presence of anaerobic or fastidious organisms that may not grow under standard culture conditions.

On the third day of admission, a second small incision was made above the right upper lip due to persistent swelling, but no pus was found. Daily dressing was continued, and her condition improved progressively.

**Figure 2:**
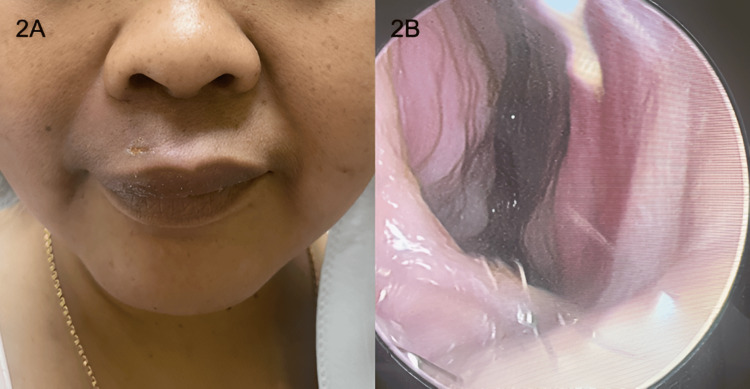
Resolution of the swelling externally (2A) and inside the nasal cavity (2B).

At her two-week follow-up, the patient showed complete recovery with no signs of residual collection (Figures 2A, 2B).

## Discussion

The two nasal cavities are separated by the nasal septum. The anterior border of the nasal cavity is the anterior naris, and posteriorly is the nasopharynx. The widened area just posterior to the naris is the nasal vestibule, which is unique because it contains hair-bearing skin but not mucosa [2]. The nasal floor, forming the lower boundary of the nasal cavity, is mainly composed of the palatine process of the maxilla anteriorly and the palatine process of the maxilla posteriorly [3]. It serves as a barrier between the nasal and oral cavities and is lined by mucosa that can be susceptible to infections under certain conditions.

Nasal floor abscesses are infrequent due to the robust vascular supply and effective anatomical defence of the nasal cavity. The exact cause of this abscess remains unclear, but possible contributing factors include minor trauma, microabrasions, or localized bacterial invasion of the nasal mucosa. Common pathogens implicated in similar infections are *Staphylococcus aureus *and *Streptococcus *species [4]. Nasal and paranasal infections are more commonly observed in individuals with predisposing factors such as preexisting dental infections, trauma, or systemic conditions like diabetes or immunosuppression [1]. Their occurrence in healthy, immunocompetent individuals without an identifiable cause is exceptionally uncommon. Nonetheless, any infection in this region, which is often referred to as the “danger triangle of the face,” requires careful management due to its unique venous drainage. The veins in this area lack valves, allowing retrograde spread of infection to the cavernous sinus, increasing the risk of cavernous sinus thrombosis.

As other infections involving the nasal cavity, the presentation of a nasal floor abscess includes localized pain and swelling, nasal obstruction once the swelling is big enough to obstruct the nasal cavity. Other systemic symptoms, such as fever and headache, may bother the patient. Our patient presented with localized swelling, tenderness, and erythema over the nasal floor extending to the upper lip, accompanied by a visible punctum with pus discharge on the nasal floor. There were no systemic symptoms such as fever or malaise, highlighting the localized nature of the infection.

Anterior rhinoscopy allows visualization of the anterior nasal cavity, including the nasal vestibule, anterior end of the inferior turbinate, nasal floor, and nasal septum. This makes it a useful bedside test for excluding a nasal septal abscess, which is a rhinologic emergency. However, as the name suggests, anterior rhinoscopy does not provide a complete view of the posterior nasal cavity. In this context, nasal endoscopy is superior, offering a more thorough and detailed examination of the nasal cavity and allowing for a better assessment of the abscess’s extent.

A computed tomography (CT) scan is valuable in assessing the extent of a nasal abscess, its involvement of surrounding structures, and potential complications such as bony erosion [5]. It also aids in ruling out an odontogenic origin by identifying the offending tooth, if present. In this case, the presence of an obvious punctum and visible pus allowed for a clinical diagnosis, bypassing the need for imaging studies. Additionally, an assessment by the dental team excluded a dental cause for the abscess. Prompt surgical intervention was performed through incision and drainage, a crucial step in preventing complications such as facial cellulitis or intracranial extension. The patient was also treated with broad-spectrum antibiotics targeting common pathogens, which facilitated effective resolution of the infection.

Similar to the management of a nasal septal abscess, appropriate treatment typically involves incision and drainage of the abscess, thorough irrigation of the cavity, administration of intravenous antibiotics, and adequate pain relief [1]. Postoperatively, the placement of a small Penrose drain or packing of the abscess cavity with diluted povidone-iodine-soaked ribbon gauze may be considered to prevent reaccumulation of pus. The Penrose drain is generally removed once there is a reduction in pus, typically around day three to day four after surgical drainage. Alternatively, if the surgeon opts for packing without a drain, daily dressing should be performed with replacement of the nasal packing during each dressing session.

## Conclusions

This report documents the first known case of a nasal floor abscess in an immunocompetent individual, highlighting the need for clinicians to consider rare anatomical sites for abscess formation in patients presenting with localized nasal swelling. The successful outcome in this case underscores the importance of prompt surgical drainage and appropriate antibiotic therapy, even in the absence of imaging. This case adds to the understanding of unusual abscess presentations and emphasizes the need for clinical vigilance in managing such rare conditions
